# Prepare and Protect: Safer Behaviors in Laboratories and Clinical Containment Settings

**DOI:** 10.3201/eid2712.211866

**Published:** 2021-12

**Authors:** Kristy Jennings, Christopher E. Carr

**Affiliations:** Georgia Institute of Technology, Atlanta, Georgia, USA

**Keywords:** biosafety, laboratory safety, behavior, personal protective equipment, PPE, safety culture

In the business of space exploration, it is said that the best spacecraft is the one that is on the rocket. Similarly, the best standard operating procedure is the one that produces the desired outcome and can be, and is, followed. By applying universal human behaviors and making safety engaging, in Prepare and Protect: Safer Behaviors in Laboratories and Clinical Containment Settings, the author, Sean G. Kaufman, encourages the reader to think about and promote biosafety in new, significant ways (Figure). 

**Figure:**
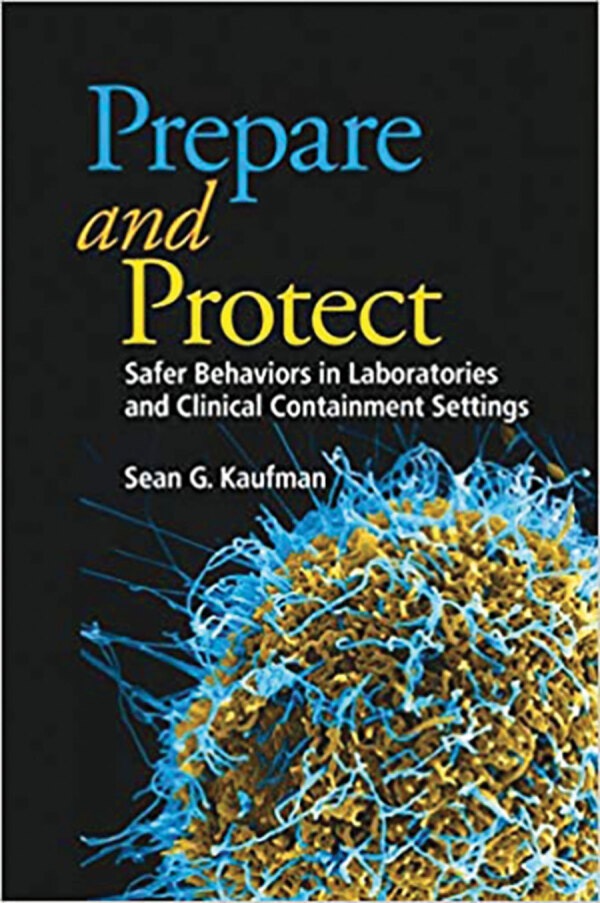
Prepare and Protect: Safer Behaviors in Laboratories and Clinical Containment Settings

Kaufman speaks to his readers in an approachable manner, taking us on an honest journey on which he asks us to rethink many preconceived ideas about what truly makes us safe or unsafe in the laboratory. Breaking down near misses, accidents, and disasters into their most basic components to see where things went wrong and why, Kaufman helps the reader begin to see patterns emerge. Memorable lessons include the impact of safety culture, the importance of meaningful risk assessment, human risk factors, and the ways biosafety professionals can best serve their programs. 

The book begins with basic biosafety principles such as containment, risk mitigation, and the primary controls of safety, always presented with interesting and important discussion and nuance. For example, in this way the author presents a proposal for clinical containment levels, thoughts on risk assessment as a living process, and the concept that it is critical to teach the workforce why they are asked to do something. We are also introduced to the intriguing subject of human risk factors, where we discover how our mental, physical, and emotional states, capabilities, institutional culture, and experiences all affect safety when working in the laboratory. This chapter is a pleasure, highlighted by stories about behavioral evolution and the dangers of appealing to authority. 

With the same unique insight and fresh approaches, later chapters consider effective plans, emergency preparedness and response, and standard operating procedures to address these challenges. We also read about the critical impact on safety culture of accountable leadership. One important contribution of note is that personal protective equipment must be matched with a corresponding and effective standard operating procedure for doffing, exemplified by the beaking method of glove removal. Some chapters could possibly have been reordered or combined to feel more intuitive, but their conciseness makes each topic easier to process. First-person narratives included are often compelling and give insight into personal journeys of biosafety practitioners, complementing didactic content with real-world experience and insight. 

The ongoing pandemic illustrates how the lessons in this book apply far beyond the lab, underscoring the extent to which we all contribute to biosafety. Aspiring, new, and experienced biosafety practitioners will enjoy this timely, informative, and engaging journey, one that can lead us to safer labs and better health for all. Above all, Kaufman reminds us to treat each other with kindness, respect, and dignity in our journeys towards safer outcomes. 

